# *Leucocalocybe mongolica* inoculation enhances rice growth by reallocating resources from flavonoid defense to development via MYB/bHLH/WRKY networks

**DOI:** 10.3389/fpls.2025.1653445

**Published:** 2025-09-03

**Authors:** Mingzheng Duan, Mei Wang, Fuhan Wei, Sirui Han, Zhifang He, Dong Hu, Qiuyue Ran, Xiande Duan, Shunqiang Yang, Muhammad Junaid Rao

**Affiliations:** ^1^ Key Laboratory of Edible Fungi Resources Innovation Utilization and Cultivation, College of Agronomy and Life Sciences, Zhaotong University, Zhaotong, China; ^2^ National Key Laboratory for Development and Utilization of Forest Food Resources, Zhejiang A & F University, Hangzhou, Zhejiang, China

**Keywords:** *Leucocalocybe mongolica*, flavonoid metabolism, plant-microbe interaction, MYB/bHLH/WRKY transcription factors, stress resilience, biofertilizer, rice growth, sustainable agriculture

## Abstract

The relationship between plants and beneficial fungi offers a sustainable approach to enhance crop productivity and stress resilience. This study investigated the effects of *Leucocalocybe mongolica* strain LY9 on rice (*Oryza sativa* L.) growth, flavonoid metabolism, and transcriptional regulation. Rice plants treated with varying concentrations of LY9-transformed soil (10%, 30%, and 50%) exhibited significant improvements in phenotypic traits, including increased tiller numbers, shoot length (989 mm), and root length (518 mm), alongside elevated chlorophyll content, indicating enhanced photosynthetic efficiency. However, total flavonoid content decreased at the highest LY9 concentration, suggesting a metabolic trade-off between growth promotion and secondary metabolite production. Transcriptomic analysis revealed dose-dependent modulation of MYB, bHLH, and WRKY transcription factor genes such as *Os04g0605100-WRKY68* and *Os05g0553400-R2R3MYB84*, while metabolomic profiling identified selective upregulation of stress-responsive flavonoids, such as chalcones (e.g., 2’,4’-dihydroxy-2,3’,6’-trimethoxychalcone and naringenin chalcone) and isoflavones (e.g., prunetin), while flavones were predominantly suppressed. Pearson correlation analyses underscored negative associations between flavonoid levels and growth traits, highlighting LY9’s role in reallocating resources from defense to growth. These findings demonstrate that LY9 enhances rice productivity by modulating flavonoid metabolism and transcriptional networks, offering insights into sustainable agricultural practices for stress resilience. Additionally, the study underscores the potential of LY9 as a biofertilizer to optimize rice growth while maintaining stress resilience through targeted metabolic adjustments.

## 1 Introduction

Rice (*Oryza sativa* L.) stands as one of the most crucial staple cereal crops worldwide, feeding more than half of the global population (Asma et al., 2023). With the ever-increasing demand for food security and sustainable agricultural practices, researchers have turned their attention to innovative approaches to enhance rice productivity without compromising environmental integrity. Among these approaches, the utilization of beneficial soil microorganisms has emerged as a promising strategy to improve plant growth, stress tolerance, and nutritional quality (Das et al., 2022). Fungal symbionts, associated with fairy ring fungi groups, have garnered significant interest due to their ability to enhance soil fertility and promote plant growth through complex biochemical interactions (Duan et al., 2022b; Wang et al., 2022b).

Flavonoids represent a diverse class of phenolic compounds that play pivotal roles in plant physiology, including UV protection, antioxidant activity, and defense against pathogens and herbivores (Rao et al., 2018; Rao and Zheng, 2025). Additionally, flavonoids exhibit significant variability in their composition and concentration across plant species, tissues, and developmental stages, reflecting their adaptive responses to environmental cues such as light, nutrient availability, and biotic stress (Rao et al., 2022b, a; Shen et al., 2022). Among different flavonoid subclasses, flavones and flavonols exhibit remarkable capacity to reduce oxidative stress caused by different pathogens and abiotic stresses. They are also involved in signaling pathways that regulate plant-microbe interactions, such as nodulation in legumes and mycorrhizal associations (Das et al., 2024). Despite their importance, the biosynthesis of flavonoids is energetically costly, and their overaccumulation can divert resources away from growth-related processes, leading to potential trade-offs between defense and productivity (Ghitti et al., 2022; Wang et al., 2022a).

The MYB, bHLH, and WRKY transcription factors (TFs) play pivotal roles in regulating flavonoid biosynthesis, orchestrating plant responses to environmental cues and microbial interactions (Wu et al., 2020; Peng et al., 2021; Zhang et al., 2023; Wang et al., 2024; Duan et al., 2025). These TFs form dynamic regulatory networks, often modulating flavonoid pathway genes, while WRKY TFs integrate stress and symbiosis signals (Wu et al., 2020; Zhang et al., 2023; Wang et al., 2024). Tomato (*Solanum lycopersicum*) R2R3 MYB gene SlMYB72 modulates coloration by directly targeting genes in chlorophyll (protochlorophyllide reductase, Mg-chelatase H subunit), and flavonoid biosynthesis (chalcone synthase), with its downregulation causing uneven pigmentation and altered metabolite levels (Wu et al., 2020). Our previous study showed that *Leucocalocybe mongolica* (LM) fungus significantly improves rice growth parameters (Duan et al., 2025), yet the underlying mechanisms governing their influence on flavonoid metabolism remain unexplored. In mutualistic symbioses, such as with fungal strain, these TFs may reallocate resources from defense (e.g., flavones) to growth-promoting metabolites, as observed in plant-fungal interactions (Das et al., 2024).

Fairy ring fungi are known for their unique growth patterns in soil and their ability to transform soil properties, leading to enhanced nutrient availability for plants (Yang et al., 2019). These fungi establish symbiotic relationships with plant roots, facilitating nutrient uptake, particularly nitrogen and phosphorus, while also modulating plant secondary metabolism (Duan et al., 2022b, a). Fairy ring fungi, like *Marasmius oreades*, induce distinct metabolic profile shifts in surrounding grasses, suggesting these metabolites may influence the ring’s characteristic zone of stimulated growth or inhibition (Bonanomi et al., 2013; Zotti et al., 2025). Recent studies have highlighted the potential of fungal strains isolated from fairy ring fungi to act as biofertilizers, offering a sustainable alternative to chemical fertilizer inputs (Wang et al., 2022b). However, the mechanisms underlying their growth-promoting effects, particularly their influence on flavonoid metabolism, a key component of plant defense and development, remain poorly understood.

Extensive field surveys investigating diverse fairy ring formations have revealed a distinctive ecological phenomenon dominated by LM, a wild edible mushroom with unique genetic characteristics (Duan et al., 2021). Previous research indicates that due to specific evolutionary gene loss, LM exists exclusively in symbiotic relationships within fairy ring ecosystems, particularly those found throughout the Inner Mongolia steppe region of China. These fairy ring formations exhibit remarkable capacity to enhance the productivity of native pasture species, notably *Leymus chinensis*, without supplementation of chemical fertilization (Duan et al., 2021, 2022a). The growth enhancement of plants is particularly significant within the DARK zone of these fairy rings, characterized by intensified leaf coloration and substantially increased biomass compared to control areas beyond ring boundaries (Duan et al., 2022b). Among the fungal species examined, *Leucocalocybe mongolica* presents exceptional interest due to its substantial plant growth promotion capabilities, although its potential applications for agricultural crops such as rice require further scientific investigation (Duan et al., 2021, 2022a, 2022b; Wang et al., 2022b; Zotti et al., 2025).

LM is a basidiomycete fungus that forms characteristic fairy rings in Asian grasslands, reveals significant potential for enhancing soil fertility and promoting plant growth without application of chemical fertilization (Yang et al., 2019; Duan et al., 2022b, a). We have successfully isolated and cultured a microbial strain, designated LY9, from the fruiting bodies of *L. mongolica*. Our research involves a soil-based fermentation substrate to simulate the plant growth-promoting effects observed in natural *L. mongolica* fairy ring ecosystems. Given the taxonomic relationship between rice and *Leymus chinensis* within the Poaceae family and their similar morphological characteristics, this study aims to investigate the efficacy of the LY9 strain as a biostimulant or biofertilizer on rice phenotypic traits and flavonoid metabolism. Understanding how microbial symbionts like LM strain LY9 modulate flavonoid pathways and MYB, bHLH, and WRKY genes could provide valuable insights into optimizing rice growth while maintaining or even enhancing stress resilience. We hypothesized that LY9 would enhance rice growth by modulating transcriptional factor genes and flavonoid biosynthesis, leading to a reallocation of resources from defense to growth. To test this hypothesis, we conducted a dose-response experiment using varying concentrations of LY9-transformed soil (10%, 30%, and 50%) and assessed changes in transcriptional factor genes, tillering, shoot and root growth, chlorophyll content, and flavonoid profiles. We also performed correlation analyses to explore relationships between growth traits, genes, and flavonoid accumulation.

## 2 Results

### 2.1 Effects of LY9 on rice phenotypic traits and total flavonoids contents

**Figure 1:**
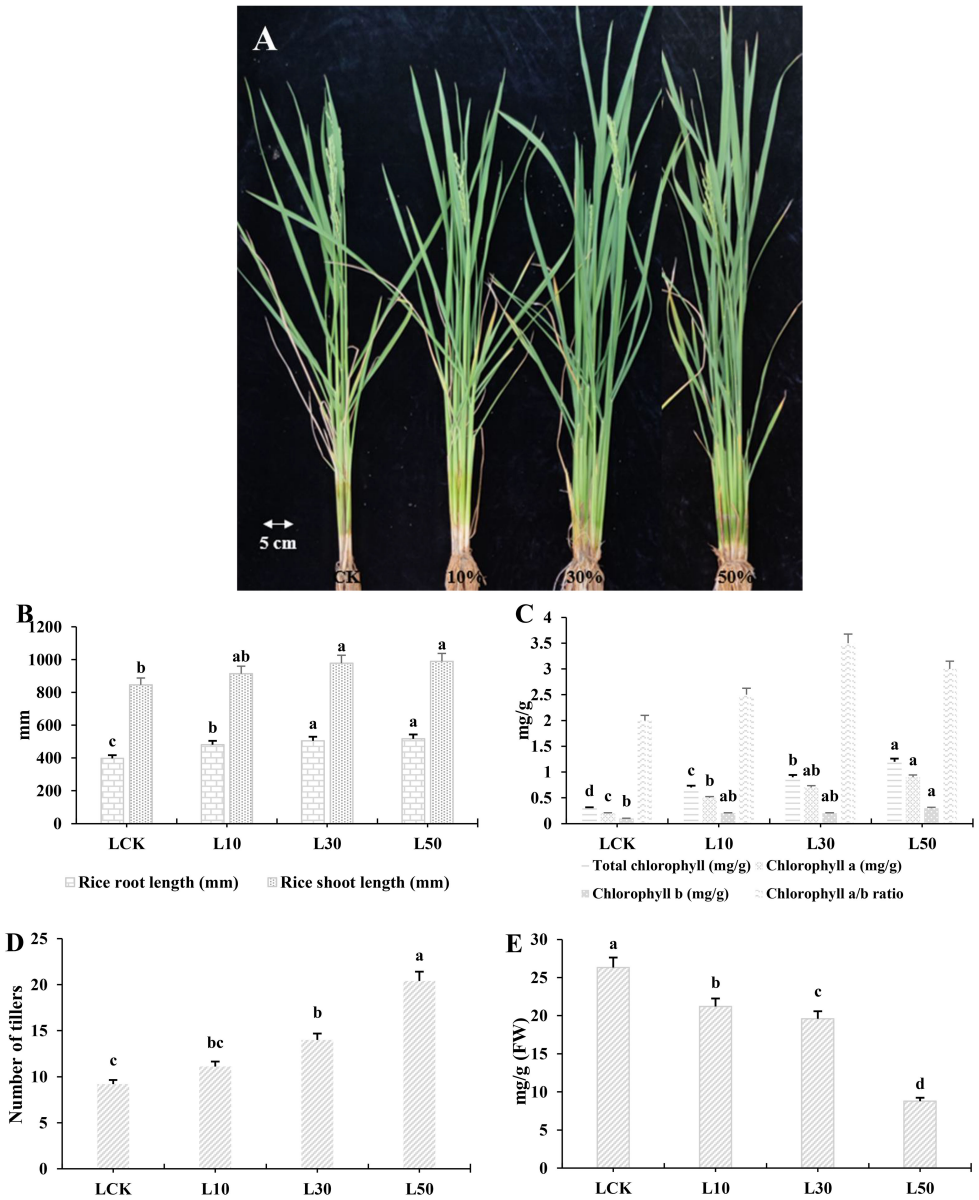
Rice phenotype, photosynthesis pigments, and flavonoid contents in leaves. **(A)** The phenotype of rice grown under three different treatments of LY9-strain and control. The control rice showed less tillering while the LY9-treated rice showed dark green leave phenotype with a greater number of tillers. **(B)** Photosynthesis pigments. **(C)** Growth parameters (root and shoot lengths in millimeters). **(D)** Number of tillers. **(E)** Total flavonoid content. Each value represented in the table is the mean of three independent biological replicates. Least significant difference (LSD) test (p< 0.05) to determine the statistical differences among LY9-treatment and CK, denoted by different lowercase letters (a, b, c). FW, fresh weight; L, Leaf; L10, L30, L50, Leaves of rice harvested from 10%, 30%, and 50% of LY9 treatment.

The study investigated the impact of the microbial strain LY9, isolated from the fruiting body of *Leucocalocybe mongolica* (LM), on rice phenotypic traits and flavonoid metabolism. The **Supplementary Table (S1)** represents the comprehensive analysis of 74 compounds which are further classified into subcategories such as 40 flavones, 21 flavonols, 4 flavanones, 3 Chalcones, 2 isoflavones, 2 other flavonoids, and one flavanonols, detailing their mass spectrometry characteristics, and quantitative measurements across different LY9 treatments. Each flavonoid is identified by its precursor ion (Q1) and product ion (Q3) masses, molecular weight, and chemical formula, alongside its ionization mode (**Supplementary Table S1**). Notably, significant variations in abundance are observed across LY9-treatments (**Supplementary Table S1**). Rice plants grown under the LY9-treated soil showed darker green leaves and significantly greater tiller phenotypes compared to controls (without-LY9) (**Figure 1A**). The phenotypic differences underscored LY9’s role in improving vegetative growth, photosynthesis efficiency, and tillering capacity (**Figure 1A**).

Regarding rice growth, both root and shoot lengths improved with increasing LY9 concentrations, achieving maximum values at 50% LY9 (518 mm and 989 mm, respectively), reflecting enhanced nutrient uptake and plant vigor (**Figure 1B**). Chlorophyll content (a, b, and total) and chlorophyll a/b ratio increased progressively with higher concentrations of LY9, indicating improved photosynthetic efficiency (**Figure 1C**). The number of tillers increased significantly with higher concentrations of 10%, 30%, and 50% LY9 treatments (11.1, 14.0, and 20.4 tillers, respectively) than control group (9.2 tillers) (**Figure 1D**). This indicates that LY9 promotes tillering, which is crucial for rice yield. However, total flavonoid content in rice leaves displayed an inverse relationship with LY9 concentration, declining from 26.3 mg/g in control to 8.8 mg/g at 50% LY9 (**Figure 1E**). This reduction suggests that while LY9 promotes soil fertility and plant growth, it may suppress flavonoid biosynthesis, possibly due to diminished oxidative stress under improved nutrient conditions. Overall, LY9 enhances chlorophyll synthesis in rice but decreased in flavonoid content highlights a potential trade-off between growth promotion and secondary metabolite production (**Figures 1B-E**).

### 2.2 MYB, bHLH, and WRKY transcriptomic profiling in rice leaves

**Figure 2:**
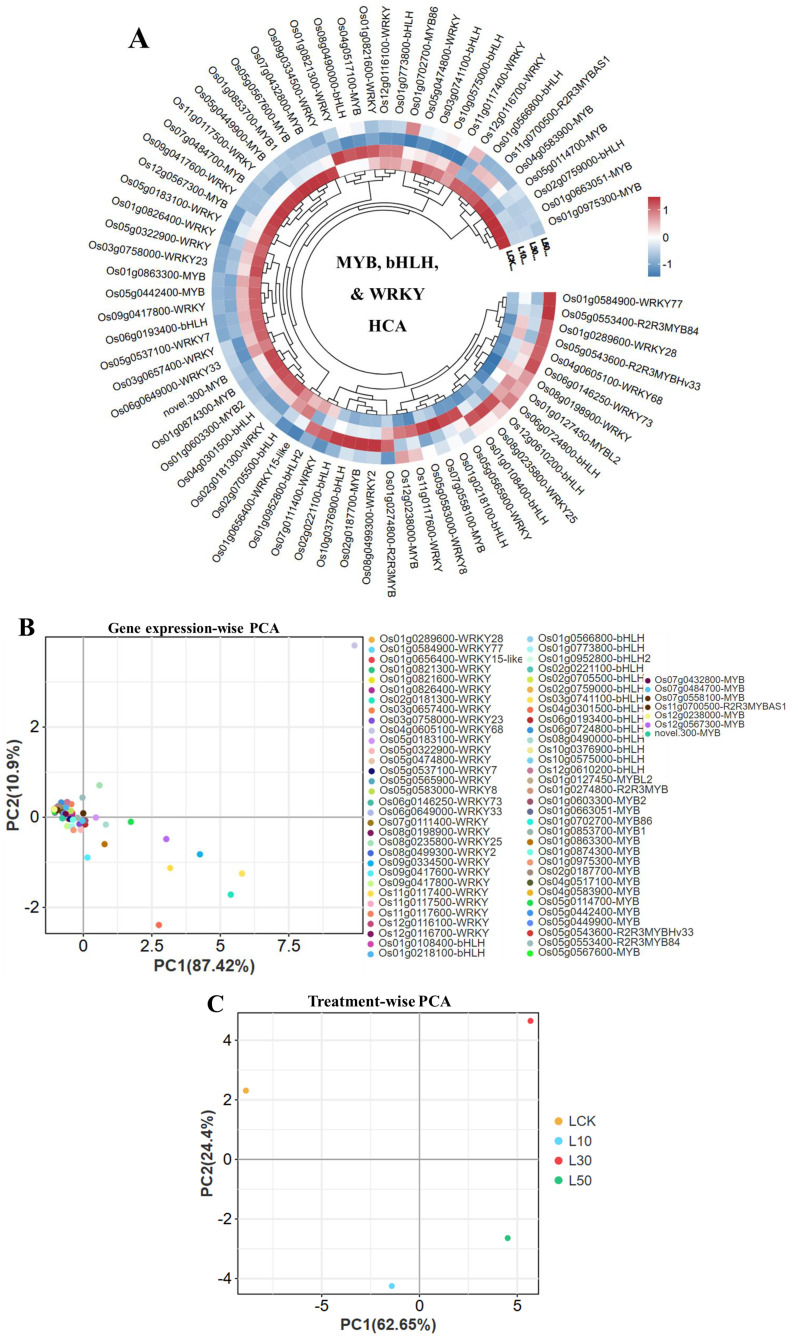
Transcriptional dynamics of rice leaves in response to LY9 treatment. **(A)** Hierarchical clustering analysis (HCA) of WRKY, bHLH, and MYB transcription factors across LY9 concentrations (LCK: control, L10: 10%, L30: 30%, L50: 50%). **(B)** Gene expression-wise principal component analysis (PCA) highlights the dominant role of WRKY, bHLH, and MYB families in driving transcriptional segregation. **(C)** PCA of treatment groups shows clear separation among control and LY9 treatment.

Our transcriptome analysis revealed systematic changes in rice transcription factor expression under LY9 treatment (**Figure 2**). Hierarchical clustering showed distinct patterns among MYB, bHLH, and WRKY gene families, with WRKY68 (Os04g0605100) exhibiting particularly strong dose-dependent upregulation (92.45 to 227.13 FPKM), while WRKY15-like (Os01g0656400) and another WRKY gene were suppressed at higher LY9 concentrations (**Figure 2A**). The bHLH and MYB families showed divergent responses - while most members were downregulated, Os06g0724800 (bHLH) and R2R3MYB84 (Os05g0553400) were notably upregulated. Principal component analysis confirmed these trends, with PC1 explaining 87.42% (**Figure 2B**) and 62.65% (**Figure 2C**) of variance and clearly separating treatment groups (**Figures 2B, C**). Controls clustered distinctly from LY9-treated samples, with 30% LY9 showing the most pronounced transcriptomic shift. These results demonstrate that LY9 induces a complex, dose-dependent reprogramming of transcriptional networks, where WRKY genes dominate early responses while MYB/bHLH genes modulate later adaptations.

### 2.3 Flavonoid profiling in rice leaves

**Figure 3:**
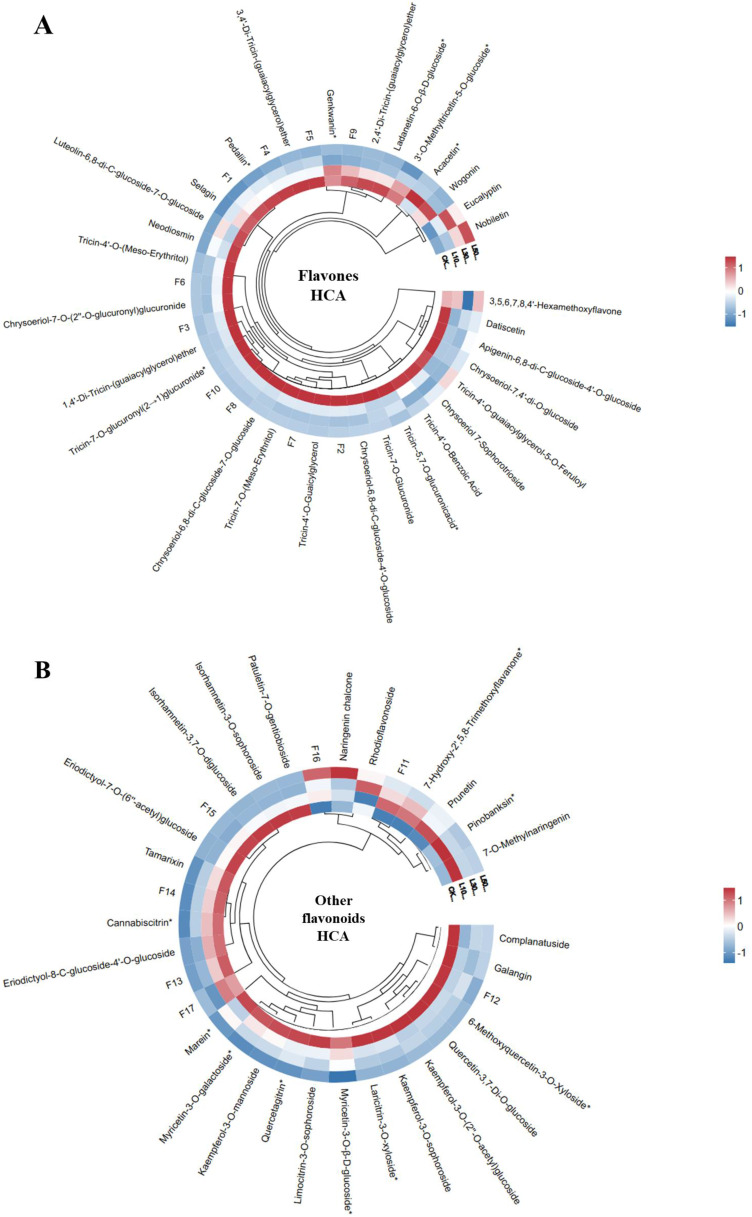
Hierarchical Cluster Analysis (HCA) of flavonoid profiles in rice leaves under control and LY9 inoculation treatments. CK, control L10, L30, L50;, Leaves of rice harvested from 10%, 30%, and 50% of LY9 treatment. **(A)** Flavone HCA, F1-F10 names consistent to **Supplementary Table S2**, and **(B)** Other flavonoids HCA, F1-F17 names corresponding to **Supplementary Table S3**. Rows represent individual flavonoid compound while the columns signify different treatments. The red color indicates high abundance whereas blue color specifies low abundance of flavonoid.

The HCA of flavones revealed distinct metabolic restructuring in the rice leaves grown under different concentrations of LY9 (**Figure 3A**). Flavones clustered into two major groups: (1) C-glycosylated derivatives (e.g., Luteolin-6,8-di-C-glucoside-7-O-glucoside) and (2) O-glycosylated/methoxylated compounds (e.g., Tricin-4’-O-(Meso-Erythritol)) (**Figure 3A**). Notably, tricin-related flavones formed a tight subcluster, suggesting conserved biosynthetic regulation. Nobiletin and eucalyptin flavones increased in response to LY9-treatment and all other flavones showed a decreasing trend as the concentration of LY9-treatment increases (L10, L30, L50) than control (**Figure 3A**). The suppression or downregulation of most flavones (**Figure 3A**) aligns with the total flavonoid contents decreased in LY9-treated samples (**Figure 1E**), reflecting a trade-off between growth promotion and defense metabolite production.

HCA of other flavonoids (such as flavonols, chalcones, flavanones, flavanonols, and isoflavones) also showed LY9-treatment-specific modulation (**Figure 3B**). Chalcones (e.g., 2’,4’-Dihydroxy-2,3’,6’-Trimethoxychalcone, naringenin chalcone) and isoflavones (e.g., Prunetin) were upregulated and clustered separately from suppressed flavonols (e.g., Kaempferol-3-O-sophoroside) (**Figure 3B**). The dose-dependent divergence of L50 samples suggests LY9 preferentially enhances stress-responsive subclasses (e.g., chalcones, 6.47-fold increase in L10) while repressing others, potentially redirecting resources to growth (**Table 1**). LY9-grown rice leaves showed systematically suppression of flavones but induces chalcones/isoflavones, suggesting targeted metabolic optimization.

**Table 1:** Rice leaves metabolites significantly altered in response to LY9 (L10 vs CK).

Serial no.	Compounds	Class	VIP	P-value	Fold change	Log_2_FC	Type
1	1,4’-Di-Tricin-(guaiacylglycerol)ether	Flavones	1.34	0.00	0.30	-1.71	down
2	2’,4’-Dihydroxy-2,3’,6’-Trimethoxychalcone*	Chalcones	1.33	0.01	6.47	2.69	up
3	3,5,7,2’-Tetrahydroxyflavone; Datiscetin	Flavones	1.32	0.00	0.46	-1.12	down
4	3,5,7-Trihydroxyflavanone (Pinobanksin)*	Flavanonols	1.18	0.01	2.28	1.19	up
5	4-C-Glucose-1,3,6-trihydroxy-7-methoxyxanthone	Flavonoids	1.25	0.02	0.37	-1.44	down
6	7-Hydroxy-2’,5,8-Trimethoxyflavanone*	Flavanones	1.30	0.01	5.50	2.46	up
7	7-O-Methylnaringenin	Flavanones	1.26	0.07	107.38	6.75	up
8	Chrysoeriol-6,8-di-C-glucoside-4’-O-glucoside	Flavones	1.33	0.00	0.49	-1.02	down
9	Chrysoeriol-6,8-di-C-glucoside-7-O-glucoside	Flavones	1.29	0.01	0.43	-1.22	down
10	Chrysoeriol-7,4’-di-O-glucoside	Flavones	1.30	0.01	0.49	-1.03	down
11	Complanatuside	Flavonols	1.26	0.01	0.47	-1.10	down
12	Diosmetin-7-O-Neohesperidoside (Neodiosmin)	Flavones	1.19	0.01	0.50	-1.00	down
13	Kaempferol-3-O-(2’’-O-acetyl)glucoside	Flavonols	1.23	0.01	0.48	-1.07	down
14	Prunetin (5,4’-Dihydroxy-7-methoxyisoflavone)	Isoflavones	1.27	0.04	36.48	5.19	up
15	Quercetin-3,7-Di-O-glucoside	Flavonols	1.29	0.01	0.46	-1.11	down
16	Tricin–5,7-O-glucuronicacid*	Flavones	1.32	0.02	0.38	-1.39	down
17	Tricin-4’-O-Guaicylglycerol	Flavones	1.33	0.00	0.43	-1.21	down
18	Tricin-4’-O-guaiacylglycerol-5-O-Feruloyl	Flavones	1.33	0.01	0.50	-1.00	down
19	Tricin-5-O-guaiacylglycerol-ether-4’-O-glucoside	Flavones	1.19	0.09	0.40	-1.34	down
20	Tricin-7-O-(Meso-Erythritol)	Flavones	1.34	0.00	0.44	-1.18	down
21	Tricin-7-O-Glucuronide	Flavones	1.33	0.01	0.33	-1.59	down
22	Tricin-7-O-glucuronyl(2→1)glucuronide*	Flavones	1.33	0.00	0.38	-1.40	down

‘*’ isomers.

### 2.4 Principal component analysis of rice leaves

**Figure 4:**
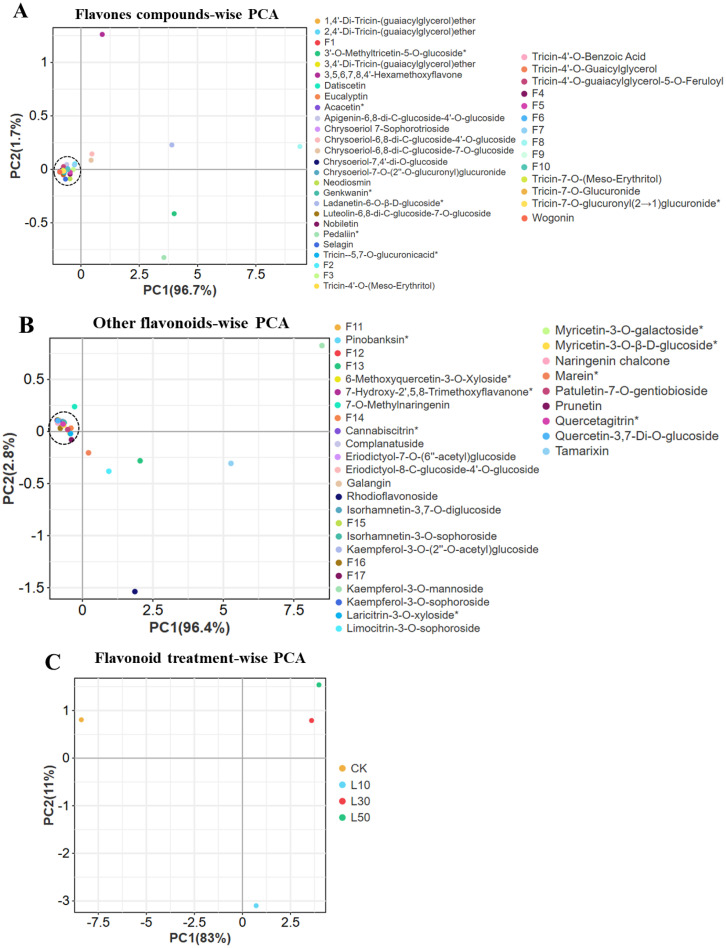
Principal component analysis (PCA) of flavonoid compounds in rice Leaves under Control and LY9 Inoculation Treatments. CK, control; L10, L30, L50, Leaves of rice harvested from 10%, 30%, and 50% of LY9 treatment. **(A)** Flavone compound-wise PCA, **(B)** Other flavonoids compound-wise PCA, and **(C)** Flavonoid treatment-wise PCA.

Our principal component analysis revealed systematic, dose-dependent changes in rice flavonoid profiles under LY9 treatment (**Figure 4**). For flavones (**Figure 4A**), PC1 (96.7% variance) showed strong separation by treatment intensity, with compounds like 3’-O-methyltricetin-5-O-glucoside and tricin derivatives exhibiting the most pronounced responses. Similar patterns emerged for other flavonoids (**Figure 4B**), where PC1 (96.4% variance) primarily differentiated treatments through glycosylated forms such as kaempferol-3-O-mannoside and quercetin derivatives. The comprehensive flavonoid analysis (**Figure 4C**) demonstrated progressive metabolic shifts along PC1 (83% variance), with control samples (rich in flavones) clearly separating from LY9-treated groups. The 50% LY9 treatment (L50) showed maximal flavonoid suppression, positioned farthest from controls. Notably, we observed class-specific responses: chalcones (naringenin chalcone) and isoflavones (prunetin) increased significantly, while most flavones and flavonols decreased. Glycosylated compounds formed distinct clusters, suggesting modified glycosylation pathways under LY9 exposure. These results demonstrate that LY9 treatment systematically reshapes flavonoid metabolism in rice leaves, with both suppression and selective induction of specific subclasses.

### 2.5 Differential flavonoid responses to LY9 treatment in rice leaves

**Figure 5:**
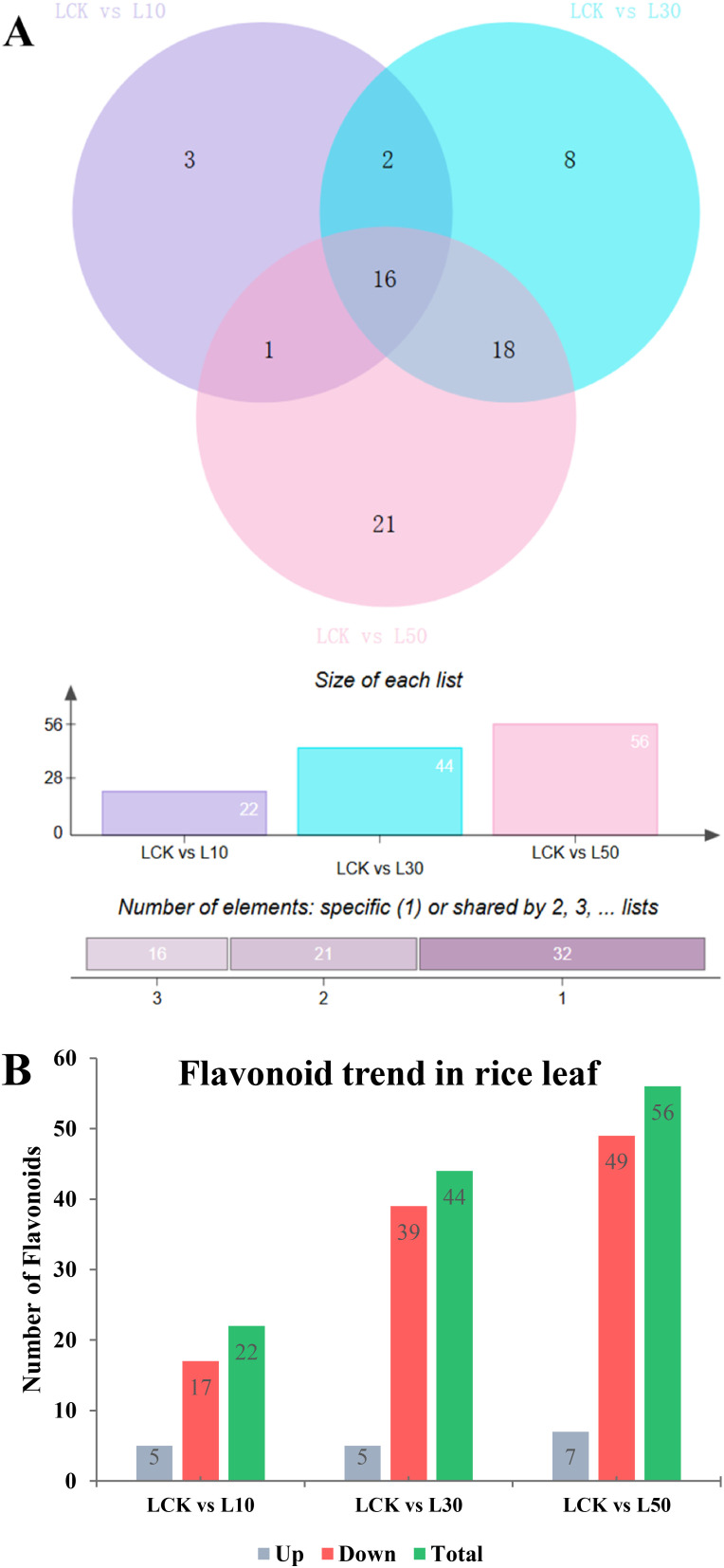
Differential flavonoid accumulation in rice leaves under varying concentrations of LY9 treatment. **(A)** Venn diagrams illustrate the number of unique and shared flavonoids altered in rice leaves under different LY9 treatments compared to the control (LCK). The top panel shows pairwise comparisons (LCK vs L10, LCK vs L30, LCK vs L50), with numerical values indicating the size of each list (number of altered flavonoids). The bottom panel categorizes the number of flavonoids as unique to one treatment compared to LCK. **(B)** Bar graph summarizing the trend of flavonoid accumulation in rice leaves under LY9 treatments. The graph compares the LCK with treatments (L10, L30, L50) and categorizes flavonoids as upregulated (Up), downregulated (Down), or total altered flavonoids (Total).

Venn diagram analysis (**Figure 5A**) revealed that the LCK vs. L10 comparison showed 3 uniquely altered flavonoids in L10, while 2 and 1 flavonoids were shared with other treatments or unique to other comparisons, respectively. In contrast, the LCK vs. L50 comparison displayed the largest number of altered flavonoids (56), with 21, 18, 16 and 1 showing unique or shared changes, and 21 flavonoids uniquely altered in L50, suggesting a dose-dependent response (**Figure 5A**). Moreover, 16 flavonoids were commonly altered across all three treatments, while 3, 8, and 21 were unique to individual treatments, highlighting conserved metabolic responses to LY9 exposure.

The bar graph showed the trends in flavonoid accumulation, categorizing changes as upregulated, downregulated, and total altered compounds (**Figure 5B**). Higher LY9 concentrations, particularly L50, induced greater 65 altered flavonoids including 49 downregulated and 7 upregulated, likely as a stress-adaptive mechanism. Downregulated flavonoids may reflect the suppression of specific metabolic pathways under LY9-induced stress, with L50 exhibiting the most significant disruption in flavonoid metabolism.

### 2.6 Flavonoids significantly different in response to LY9 (L10 vs CK)

In the L10 treatment, 22 metabolites were significantly altered compared to the control. Among these, 5 metabolites were upregulated, while 17 were overregulated (**Figure 5B** and **Table 1**). The upregulated metabolites included chalcones and flavanones, such as 2’,4’-Dihydroxy-2,3’,6’-Trimethoxychalcone (Fold Change: 6.47, Log_2_FC: 2.69) and 7-O-Methylnaringenin (Fold Change: 107.38, Log_2_FC: 6.75). Downregulated metabolites were predominantly flavones, such as 1,4’-Di-Tricin-(guaiacylglycerol)ether (Fold Change: 0.30, Log_2_FC: -1.71) and tricin-7-O-Glucuronide (Fold Change: 0.33, Log2FC: -1.59). These results suggest that even at a low concentration, LY9 significantly modulates flavonoid metabolism, particularly by enhancing the production of certain chalcones and flavanones while suppressing several flavones (**Table 1**).

### 2.6 Flavonoids significantly altered in response to LY9 (L30 vs CK)

In the L30 treatment, 44 metabolites were significantly altered, with 5 upregulated and 39 downregulated (**Figure 5B** and **Table 2**). Similar to the L10 treatment, the upregulated metabolites included 2’,4’-Dihydroxy-2,3’,6’-Trimethoxychalcone (Fold Change: 4.62, Log2FC: 2.21) and prunetin (Fold Change: 17.31, Log2FC: 4.11). Downregulated metabolites were again predominantly flavones, such as 1,4’-Di-Tricin-(guaiacylglycerol)ether (Fold Change: 0.19, Log2FC: -2.43) and tricin-7-O-Glucuronide (Fold Change: 0.34, Log2FC: -1.58) (**Table 2**). The increased number of downregulated metabolites at this concentration indicates a more pronounced suppression of flavone biosynthesis, while the upregulation of specific chalcones and isoflavones suggests a shift in metabolic pathways favoring these compounds.

**Table 2:** Rice leaves metabolites significantly different in response to LY9 (L30 vs CK).

Serial no.	Compounds	Class	VIP	P-value	Fold change	Log_2_FC	Type
1	1,4’-Di-Tricin-(guaiacylglycerol)ether	Flavones	1.30	0.00	0.19	-2.43	down
2	2’,4’-Dihydroxy-2,3’,6’-Trimethoxychalcone*	Chalcones	1.23	0.07	4.62	2.21	up
3	2,4’-Di-Tricin-(guaiacylglycerol)ether	Flavones	1.29	0.00	0.41	-1.29	down
4	3,4’-Di-Tricin-(guaiacylglycerol)ether	Flavones	1.22	0.01	0.46	-1.11	down
5	3,5,6,7,8,4’-Hexamethoxyflavone	Flavones	1.30	0.00	0.00	-9.47	down
6	4-C-Glucose-1,3,6-trihydroxy-7-methoxyxanthone	Flavonoids	1.22	0.02	0.43	-1.23	down
7	5,7,4’-Trihydroxy-6,8-dimethoxyisoflavone-7-O-galactoside-glucose-rhamnose	Isoflavones	1.17	0.08	0.47	-1.10	down
8	5-Hydroxy-7,4’-dimethoxy-6,8-dimethylflavone; Eucalyptin	Flavones	1.28	0.01	2.04	1.03	up
9	7-Hydroxy-2’,5,8-Trimethoxyflavanone*	Flavanones	1.20	0.09	4.52	2.18	up
10	7-O-Methylnaringenin	Flavanones	1.14	0.10	16.54	4.05	up
11	Apigenin-6,8-di-C-glucoside-4’-O-glucoside	Flavones	1.29	0.01	0.48	-1.06	down
12	Chrysoeriol 7-Sophorotrioside	Flavones	1.19	0.01	0.43	-1.22	down
13	Chrysoeriol-6,8-di-C-glucoside-4’-O-glucoside	Flavones	1.30	0.00	0.38	-1.41	down
14	Chrysoeriol-6,8-di-C-glucoside-7-O-glucoside	Flavones	1.27	0.01	0.36	-1.48	down
15	Chrysoeriol-7,4’-di-O-glucoside	Flavones	1.27	0.02	0.49	-1.01	down
16	Chrysoeriol-7-O-(2’’-O-glucuronyl)glucuronide	Flavones	1.28	0.00	0.40	-1.32	down
17	Eriodictyol-7-O-(6’’-acetyl)glucoside	Flavanones	1.26	0.00	0.41	-1.27	down
18	Eriodictyol-8-C-glucoside-4’-O-glucoside	Flavanones	1.09	0.05	0.47	-1.10	down
19	Galangin (3,5,7-Trihydroxyflavone)	Flavonols	1.24	0.00	0.46	-1.13	down
20	Genkwanin (Apigenin 7-methyl ether)*	Flavones	1.13	0.03	0.45	-1.15	down
21	Isorhamnetin-3,7-O-diglucoside	Flavonols	1.30	0.01	0.30	-1.72	down
22	Isorhamnetin-3-O-(2’’-O-glucosyl)galactoside-7-O-glucoside	Flavonols	1.30	0.00	0.18	-2.49	down
23	Isorhamnetin-3-O-sophoroside	Flavonols	1.28	0.01	0.30	-1.74	down
24	Kaempferol-3-O-(2’’-O-acetyl)glucoside	Flavonols	1.29	0.00	0.49	-1.02	down
25	Kaempferol-3-O-mannoside(Amoenin)	Flavonols	1.29	0.00	0.46	-1.13	down
26	Patuletin-7-O-gentiobioside	Flavonols	1.26	0.04	0.43	-1.21	down
27	Pedaliin*	Flavones	1.24	0.00	0.48	-1.05	down
28	Prunetin (5,4’-Dihydroxy-7-methoxyisoflavone)	Isoflavones	1.20	0.06	17.31	4.11	up
29	Quercetin-3,7-Di-O-glucoside	Flavonols	1.21	0.01	0.44	-1.19	down
30	Selagin	Flavones	1.29	0.00	0.49	-1.02	down
31	Tricin–5,7-O-glucuronicacid*	Flavones	1.24	0.02	0.44	-1.19	down
32	Tricin-4’-O-(2’’-Sinapoyl)glucoside-5-O-glucoside*	Flavones	1.30	0.00	0.40	-1.32	down
33	Tricin-4’-O-(2’’-Sinapoyl)glucoside-7-O-glucoside	Flavones	1.26	0.00	0.44	-1.19	down
34	Tricin-4’-O-(Meso-Erythritol)	Flavones	1.30	0.00	0.38	-1.39	down
35	Tricin-4’-O-Benzoic Acid	Flavones	1.22	0.00	0.43	-1.22	down
36	Tricin-4’-O-Guaicylglycerol	Flavones	1.29	0.00	0.31	-1.69	down
37	Tricin-4’-O-guaiacylglycerol-5-O-Feruloyl	Flavones	1.28	0.00	0.50	-1.01	down
38	Tricin-5-O-(4’-O-Rhamnoside)-guaiacylglycerol-ether	Flavones	1.30	0.00	0.47	-1.09	down
39	Tricin-5-O-glucoside-7-O-(2’’-Sinapoyl)glucoside*	Flavones	1.26	0.02	0.42	-1.24	down
40	Tricin-5-O-guaiacylglycerol-ether-4’-O-glucoside	Flavones	1.19	0.08	0.33	-1.60	down
41	Tricin-7-O-(2’’-O-rhamnosyl)galacturonide	Flavones	1.22	0.00	0.50	-1.00	down
42	Tricin-7-O-(Meso-Erythritol)	Flavones	1.30	0.00	0.31	-1.70	down
43	Tricin-7-O-Glucuronide	Flavones	1.28	0.01	0.34	-1.58	down
44	Tricin-7-O-glucuronyl(2→1)glucuronide*	Flavones	1.26	0.00	0.32	-1.66	down

‘*’ isomers.

### 2.7 Flavonoids significantly changed in response to LY9 (L50 vs CK)

In the L50 treatment, 56 metabolites were significantly altered, with 7 upregulated and 49 downregulated (**Figure 5B** and **Table 3**). The upregulated metabolites included 2’,4’-Dihydroxy-2,3’,6’-Trimethoxychalcone (Fold Change: 3.52, Log2FC: 1.81) and prunetin (Fold Change: 16.55, Log2FC: 4.05). Downregulated metabolites were again predominantly flavones, such as 1,4’-Di-Tricin-(guaiacylglycerol)ether (Fold Change: 0.17, Log2FC: -2.54) and tricin-7-O-glucuronide (Fold Change: 0.33, Log2FC: -1.61). The results at this highest concentration further emphasize the consistent upregulation of chalcones and isoflavones, while flavones and other flavonoid classes are increasingly suppressed (**Table 3**).

**Table 3:** Rice leaves metabolites significantly different in response to LY9 (L50 vs CK).

Serial no.	Compounds	Class	VIP	P-value	Fold change	Log_2_FC	Type
1	1,4’-Di-Tricin-(guaiacylglycerol)ether	Flavones	1.27	0.00	0.17	-2.54	down
2	2’,4’-Dihydroxy-2,3’,6’-Trimethoxychalcone*	Chalcones	1.24	0.02	3.52	1.81	up
3	2,4’-Di-Tricin-(guaiacylglycerol)ether	Flavones	1.26	0.00	0.42	-1.26	down
4	3’,5’,5,7-Tetrahydroxy-4’-methoxyflavanone-3’-O-glucoside	Flavones	1.23	0.00	0.31	-1.67	down
5	3’-O-Methyltricetin-5-O-glucoside*	Flavones	1.21	0.00	0.40	-1.31	down
6	3,4’-Di-Tricin-(guaiacylglycerol)ether	Flavones	1.27	0.00	0.39	-1.36	down
7	4-C-Glucose-1,3,6-trihydroxy-7-methoxyxanthone	Flavonoids	1.24	0.02	0.24	-2.07	down
8	5,7,4’-Trihydroxy-6,8-dimethoxyisoflavone-7-O-galactoside-glucose-rhamnose	Isoflavones	1.17	0.06	0.40	-1.32	down
9	6-Methoxyquercetin-3-O-Xyloside*	Flavonols	1.25	0.00	0.46	-1.12	down
10	7-Hydroxy-2’,5,8-Trimethoxyflavanone*	Flavanones	1.18	0.01	2.90	1.53	up
11	7-O-Methylnaringenin	Flavanones	1.12	0.02	13.57	3.76	up
12	8,11-dimethoxy-2h-[1,3]dioxolo[4,5-b]xanthen-10-one	Flavonoids	1.16	0.01	0.42	-1.25	down
13	Cannabiscitrin*	Flavonols	1.26	0.00	0.37	-1.45	down
14	Chrysoeriol-6,8-di-C-glucoside-4’-O-glucoside	Flavones	1.27	0.00	0.42	-1.24	down
15	Chrysoeriol-6,8-di-C-glucoside-7-O-glucoside	Flavones	1.25	0.02	0.36	-1.47	down
16	Chrysoeriol-7-O-(2’’-O-glucuronyl)glucuronide	Flavones	1.22	0.00	0.42	-1.24	down
17	Diosmetin-7-O-Neohesperidoside (Neodiosmin)	Flavones	1.21	0.00	0.32	-1.64	down
18	Eriodictyol-7-O-(6’’-acetyl)glucoside	Flavanones	1.26	0.00	0.41	-1.28	down
19	Eriodictyol-8-C-glucoside-4’-O-glucoside	Flavanones	1.16	0.02	0.42	-1.24	down
20	Isorhamnetin-3,7-O-diglucoside	Flavonols	1.24	0.00	0.30	-1.76	down
21	Isorhamnetin-3-O-(2’’-O-glucosyl)galactoside-7-O-glucoside	Flavonols	1.23	0.00	0.22	-2.20	down
22	Isorhamnetin-3-O-sophoroside	Flavonols	1.25	0.01	0.31	-1.67	down
23	Kaempferol-3-O-(2’’-O-acetyl)glucoside	Flavonols	1.26	0.00	0.42	-1.27	down
24	Kaempferol-3-O-(6’’-Malonyl)glucoside-7-O-Glucoside	Flavonols	1.09	0.04	2.00	1.00	up
25	Kaempferol-3-O-mannoside (Amoenin)	Flavonols	1.27	0.00	0.20	-2.29	down
26	Kaempferol-3-O-sophoroside	Flavonols	1.27	0.00	0.48	-1.05	down
27	Ladanetin-6-O-β-D-glucoside*	Flavones	1.24	0.02	0.49	-1.02	down
28	Laricitrin-3-O-xyloside*	Flavonols	1.24	0.00	0.47	-1.10	down
29	Limocitrin-3-O-sophoroside	Flavonols	1.26	0.00	0.49	-1.02	down
30	Luteolin-6,8-di-C-glucoside-7-O-glucoside	Flavones	1.24	0.01	0.44	-1.19	down
31	Myricetin-3-O-galactoside*	Flavonols	1.27	0.00	0.44	-1.18	down
32	Myricetin-3-O-β-D-glucoside*	Flavonols	1.26	0.00	0.48	-1.05	down
33	Naringenin chalcone; 2’,4,4’,6’-Tetrahydroxychalcone	Chalcones	1.27	0.00	5.58	2.48	up
34	Nobiletin (5,6,7,8,3’,4’-Hexamethoxyflavone)	Flavones	1.19	0.01	2.88	1.53	up
35	Okanin-4’-O-glucoside (Marein)*	Chalcones	1.16	0.01	0.49	-1.04	down
36	Patuletin-7-O-gentiobioside	Flavonols	1.22	0.04	0.45	-1.16	down
37	Pedaliin*	Flavones	1.25	0.00	0.24	-2.07	down
38	Prunetin (5,4’-Dihydroxy-7-methoxyisoflavone)	Isoflavones	1.18	0.00	16.55	4.05	up
39	Quercetagetin-7-O-glucoside (Quercetagitrin)*	Flavonols	1.24	0.00	0.39	-1.36	down
40	Quercetin-3,7-Di-O-glucoside	Flavonols	1.22	0.00	0.37	-1.42	down
41	Selagin	Flavones	1.27	0.00	0.18	-2.49	down
42	Tamarixetin-3-O-glucoside (Tamarixin)	Flavonols	1.26	0.00	0.33	-1.61	down
43	Tricin–5,7-O-glucuronicacid*	Flavones	1.24	0.02	0.32	-1.62	down
44	Tricin-4’-O-(2’’-Sinapoyl)glucoside-5-O-glucoside*	Flavones	1.26	0.00	0.44	-1.18	down
45	Tricin-4’-O-(2’’-Sinapoyl)glucoside-7-O-glucoside	Flavones	1.26	0.00	0.40	-1.34	down
46	Tricin-4’-O-(Meso-Erythritol)	Flavones	1.26	0.00	0.35	-1.50	down
47	Tricin-4’-O-Guaicylglycerol	Flavones	1.27	0.00	0.33	-1.61	down
48	Tricin-4’-O-syringylglyceryl ether-5-O-glucoside*	Flavones	1.27	0.00	0.40	-1.32	down
49	Tricin-4’-O-syringylglyceryl ether-7-O-glucoside*	Flavones	1.27	0.00	0.41	-1.28	down
50	Tricin-5-O-(4’-O-Rhamnoside)-guaiacylglycerol-ether	Flavones	1.26	0.00	0.48	-1.05	down
51	Tricin-5-O-glucoside-7-O-(2’’-Sinapoyl)glucoside*	Flavones	1.24	0.03	0.45	-1.16	down
52	Tricin-5-O-guaiacylglycerol-ether-4’-O-glucoside	Flavones	1.19	0.07	0.29	-1.80	down
53	Tricin-7-O-(4’-O-Rhamnoside)-guaiacylglycerol-ether	Flavones	1.27	0.00	0.47	-1.08	down
54	Tricin-7-O-(Meso-Erythritol)	Flavones	1.26	0.00	0.35	-1.53	down
55	Tricin-7-O-Glucuronide	Flavones	1.26	0.01	0.33	-1.61	down
56	Tricin-7-O-glucuronyl(2→1)glucuronide*	Flavones	1.26	0.00	0.29	-1.81	down

‘*’ isomers.

### 2.8 Correlation analysis among phenotypic traits, flavonoids, and TFs genes

The correlation analysis between various phenotypic traits and total flavonoid content is represented (**Table 4**). The results revealed strong positive correlations with shoot length (0.84), root length (0.82), chlorophyll a content (0.97*), chlorophyll b content (0.98*), and total chlorophyll content (0.98*) (**Table 4**). This indicates that increased tillering is associated with improved shoot and root growth, as well as higher chlorophyll levels. Shoot length was strongly correlated with root length (0.97*), chlorophyll a content (0.94*), chlorophyll b content (0.91*), and total chlorophyll content (0.94*) (**Table 4**). This suggests that shoot growth is closely linked to root growth and photosynthetic capacity. Root length was significantly correlated with chlorophyll ‘a’ content (0.92*), chlorophyll b content (0.91*), and total chlorophyll content (0.92*), indicating that root growth is also associated with enhanced photosynthesis. Both chlorophyll a and b were also strongly correlated with tillering number, shoot length, and root length, indicating a positive and significant link between chlorophyll content and overall plant growth. Total flavonoid content showed significantly negative correlations with tillering number (-0.98*), chlorophyll a content (-0.96*), chlorophyll b content (-0.98*), and total chlorophyll content (-0.97*). This suggests that as flavonoid content decreases, phenotypic traits such as tillering, shoot length, and chlorophyll content improve, indicating a potential trade-off between flavonoid production and growth-related traits, under LY9-treatment.

**Table 4:** Correlation analysis among phenotypic traits and total flavonoid contents.

Variables	Tillering number	Shoot length	Root length	Chlorophyll a	Chlorophyll b	Total chlorophyll	Total flavonoid
Tillering Number	1						
Shoot length	0.84	1					
Root length	0.82	0.97*	1				
Chlorophyll a	0.97*	0.94*	0.92*	1			
Chlorophyll b	0.98*	0.91*	0.91*	0.99*	1		
Total chlorophyll	0.98*	0.94*	0.92*	0.99*	0.99*	1	
Total flavonoid	-0.98*	-0.82	-0.83	-0.96*	-0.98*	-0.97*	1

*Significantly correlated.

**Figure 6:**
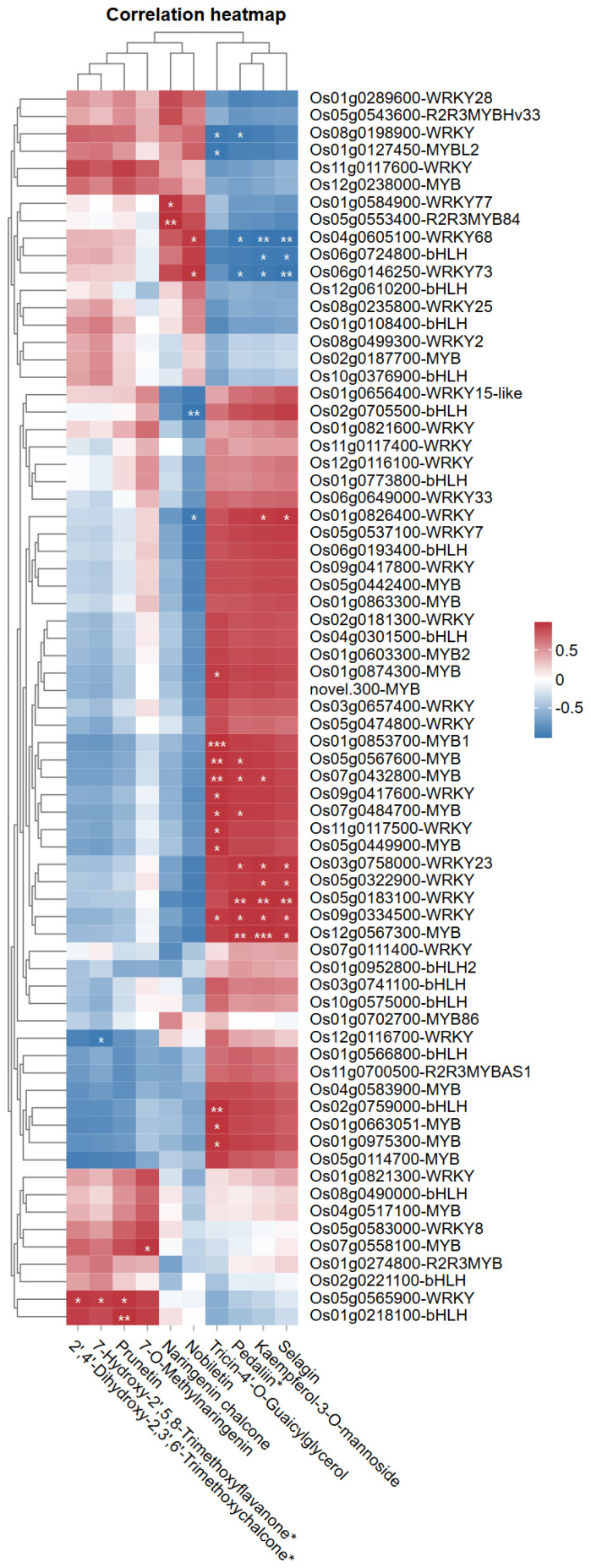
Pearson correlation heatmap of MYB, WRKY, and bHLH transcription factors expression levels with key flavonoids under LY9 treatment. Rows represent flavonoid compounds, while columns depict transcription factor genes. Pearson correlation coefficients are displayed, with red/blue indicating positive/negative associations (|r| > 0.7, student’s t-test: * P< 0.05, ** P< 0.01). Asterisks (*) mark significant associations.

The correlation heatmap illustrates the relationships between MYB, WRKY, and bHLH transcription factors expression levels and abundance of key flavonoids regulated under LY9 treatment (**Figure 6**). The heatmap reveals distinct patterns of association, with upregulated flavonoids such as prunetin (16.55-fold), 7-O-methylnaringenin (13.57-fold), naringenin chalcone (5.58-fold), and nobiletin (2.88-fold), showing strong positive correlations with specific transcription factors such as Os05g0565900-WRKY, Os01g0218100-bHLH, Os05g0553400-R2R3MYB84, and Os04g0605100-WRKY68, respectively (**Figure 6**). Additioanlly, the Os05g0565900-WRKY showed positive and significant correlation with two upregulated flavonoids (50%LY9) such as 7-hydroxy-2’,5,8-trimethoxyflavanone (2.90-fold), and 2’,4’-dihydroxy-2,3’,6’-trimethoxychalcone (3.52-fold) (**Figure 6**). Interestingly, significant and positive correlations were observed among *Os04g0605100-WRKY68* and *Os05g0553400-R2R3MYB84* with nobiletin and naringenin chalcone respectively (**Figure 6**). The transcriptomic analysis showed that the expression patterns of these two genes and the abundance of nobiletin and naringenin chalcone were significantly higher at 50% LY9 (**Figure 2A** and **Table 3**). Conversely, downregulated flavonoids like kaempferol-3-O-mannoside (-2.29-Log_2_FC), selagin (-2.49-Log_2_FC), pedaliin (-2.07-fold), and tricin-4’-O-Guaicylglycerol (-1.61-Log_2_FC) exhibit negative correlations with certain genes. Notably, MYB and WRKY genes, such as Os01g0127450-MYBL2 and Os11g0117600-WRKY, appear prominently associated with upregulated flavonoids, suggesting their potential role in enhancing flavonoid biosynthesis.

## 3 Discussion

Our study demonstrates that soil inoculation with *Leucocalocybe mongolica* strain LY9 modulates rice growth and flavonoid metabolism in a dose-dependent manner. Three key findings emerge: (1) LY9 enhances photosynthetic efficiency and tillering (**Figures 1A–D**), (2) transcriptional reprogramming of MYB/bHLH/WRKY TFs correlates with flavonoid trade-offs (**Figure 2**; **Tables 1**-**3**); and (3) selective induction of stress-responsive chalcones/isoflavones (e.g., prunetin, 36.5-fold) may compensate for suppressed flavones (**Figure 1E**). Our findings align with recent research demonstrating the growth-promoting effects of fungal symbionts (Duan et al., 2025), while offering novel insights into the metabolic trade-offs involved in these plant-microbe interactions. Our results also corroborate with the previous reports of fairy ring fungi-mediated growth-defense reallocation (Yang et al., 2019; Duan et al., 2025) but extend the paradigm by implicating specific TFs (e.g., WRKY68) in this process. The improved chlorophyll content and photosynthetic efficiency likely reflect improved nutrient availability, as commonly mediated by beneficial fungi (Das et al., 2024; Duan et al., 2025). Similar chlorophyll increment has been reported with other beneficial fungi, suggesting a common mechanism involving improved photosynthetic efficiency (Duan et al., 2022b, a).

The dose-dependent upregulation of *Os04g0605100-WRKY68* and *Os05g0553400-R2R3MYB84* genes (**Figures 2A, B**) parallels the suppression of flavones (e.g., tricin derivatives; **Tables 1**-**3**), suggesting these TFs may repress flavonoid biosynthesis pathways under LY9-induced nutrient enrichment. Notably, WRKY is a known activator/suppressor of phenylpropanoid genes in rice, while R2R3MYB genes regulate the expression of flavonoid biosynthesis genes (Wu et al., 2020; Zhang et al., 2023; Wang et al., 2024). This aligns with our observation of elevated chalcones (e.g., 2’,4’-dihydroxychalcone; **Table 1**), which are critical for stress acclimation (Duan et al., 2024a; Rao et al., 2024; Rao and Zheng, 2025). Moreover, downregulated WRKY genes (e.g., Os01g0656400-WRKY15-like) may reflect reduced stress signaling under improved nutrient conditions (Duan et al., 2022a, 2025; Zhang et al., 2023; Das et al., 2024). These findings highlight LY9’s ability to modulate TF networks, optimizing growth while retaining selective flavonoid. However, expression pattern verification and functional studies of WRKY68 and R2R3MYB84 (e.g., gene silencing/overexpression) are needed to establish causal mechanisms.

The 66% reduction in total flavonoids at 50% LY9 (**Figure 1E**) supports the “growth-defense trade-off” hypothesis, where plants under favorable conditions reduce investment in defense compounds to maximize growth (Figueroa-Macías et al., 2021; Ghitti et al., 2022; Wang et al., 2022a; Das et al., 2024), but the selective induction of chalcones/isoflavones implies a nuanced reallocation. Recent work shows that beneficial fungi often suppress flavones (defense-related) while inducing chalcones (signaling-related) to optimize resource use (Wang et al., 2022a; Das et al., 2024). This modulation parallels with previous findings, who reported selective metabolic induction in plants under beneficial microbial colonization (Ghitti et al., 2022; Wang et al., 2022a; Duan et al., 2024b). Chalcones and isoflavones are known to facilitate plant-microbe communication while providing abiotic stress tolerance (Duan et al., 2024a; Rao et al., 2024; Rao and Zheng, 2025). The flavonoid modulation could result from fungal production of signaling molecules that interact with plant metabolic pathways, as demonstrated for other microbial symbionts (Ghitti et al., 2022; Das et al., 2024; Duan et al., 2024b). Alternatively, the fungus may alter soil chemistry in ways that indirectly influence plant metabolism, consistent with known fairy ring effects on nutrient cycling (Duan et al., 2022a).In our system, this shift may reflect LY9’s dual role: enhancing growth via nutrient mobilization (Duan et al., 2025) while maintaining stress resilience through alternative metabolites (e.g., 36.48-fold increase of prunetin’s, antioxidant activity; Rao et al., 2025).

Our results showed that flavones (critical for pathogen defense; (Shen et al., 2022) were suppressed (**Tables 1**-**3**), this may reflect LY9’s prioritization of growth over constitutive defense, as seen in mycorrhizal symbioses (Ghitti et al., 2022; Wang et al., 2022a). Notably, induced chalcones/isoflavones (e.g., prunetin) likely compensate via antioxidant activity (Rao et al., 2025), mirroring strategic defense allocation in optimized crops (Wang et al., 2022a). The dose-dependent responses (**Figures 1A–C**) align with microbial hormesis, where benefits scale with dosage (Wang et al., 2022a). These findings position LY9 as a promising biofertilizer that enhances productivity without compromising stress resilience—a critical advantage for resource-limited agriculture (Das et al., 2022). Future studies should (1) validate field efficacy and (2) dissect how LY9 balances flavone suppression with alternative defense pathways.

## 4 Materials and methods

### 4.1 LY9 strain cultivation

The potato dextrose agar (PDA) medium was formulated according to standard protocols (**Supplementary File 1**). The fungal strain from the fruiting bodies of *Leucocalocybe mongolica* (designated as LY9) was subjected to cultivation until complete mycelial colonization of the medium was observed. Under aseptic conditions, mycelial fragments of the LY9 strain were excised and transferred via sterile inoculation tool onto the PDA substrate. The mycelial tissue was positioned with the active growth surface in direct contact with the nutritive medium. Inoculated plates were maintained at a constant temperature of 25 °C for a period of 20 days, during which time complete colonization of the substrate by fungal mycelia was achieved and documented (**Supplementary Figure S1**).

### 4.2 Soil preparation

Potato dextrose agar (PDA) was utilized for mycelium cultured and to establish solid-state cultures that subsequently functioned as inoculum for rice cultivation experiments. Premium quality wheat grains were subjected to thorough washing and soaked in water for 12 hours. The hydrated grains were transferred to polyethylene bags (22 cm × 45 cm) and sterilized by autoclaving at 121 °C for 60 minutes. Following thermal equilibration, the sterile grains were inoculated with LY9 culture from PDA medium and maintained under controlled conditions for 40 days until complete colonization was evident.

The substrate for growth comprised a homogeneous mixture of paddy field soil (sourced from regional arid paddy fields) and commercial peat soil (Pin’s peat soil) combined in equal proportions (1:1). This composite medium underwent sterilization within polyethylene bags equipped with inoculation ports at 121 °C for 60 minutes. Following sterilization, approximately 10g of LY9-colonized wheat grain spawn was aseptically introduced into individual bags. The inoculated substrate was maintained at ambient temperature in darkness for 60 days until comprehensive mycelial colonization was observed. This protocol generated 100 bags of LY9 transformation soil, yielding approximately 150 kg wet weight (**Supplementary Figure S2**). The fully colonized substrate exhibited uniform white appearance throughout (**Supplementary Figure S2**).

### 4.3 LY9 transformation soil and rice cultivation

We investigated the impact of LY9-transformed soil on *Oryza sativa* ssp. japonica cv. ZH11 growth utilizing an outdoor potting system. The experiment evaluated various proportions of LY9-transformed soil to assess phenotypic responses and collect tissue samples for further analysis. Rice seedlings were cultivated in outdoor containers (25 cm × 35 cm) containing mixtures of LY9-transformed soil and conventional paddy soil at concentrations of 10%, 30%, and 50%, with untransformed paddy soil (0%) serving as the control treatment (CK). Each experimental condition was implemented with 10 replicates. Plant establishment and outdoor cultivation commenced on June 15, 2023, and no supplementary fertilization was administered throughout the experimental duration.

Following approximately two months of growth, rice plants cultivated in LY9-transformed soil demonstrated pronounced growth enhancement relative to control specimens, as illustrated in **Figure 1**. To elucidate the physiological and metabolic mechanisms underlying these growth-stimulating effects, we harvested flag leaf tissues from plants grown under control, 10%, 30%, and 50% treatment conditions using rapid freezing in liquid nitrogen. These samples (with three replicates) were preserved for comprehensive analyses including chlorophyll content determination, flavonoid contents, transcriptomic analysis, and metabolomic profiling.

### 4.4 Total flavonoid contents mg/g(FW)

Flag leaves of rice were collected after cultivation in soil containing different proportions of LY9-transformed soil (control, 10%, 30%, and 50%). Three biological replicates were established for each treatment. Rice flag leaf samples (approximately 0.3 g) were accurately weighed and subjected to double reflux extraction using 60% ethanol (Chang et al., 2002). The combined filtrates were evaporated to dryness, reconstituted in 60% ethanol, and brought to a final volume of 25 mL. For flavonoid analysis, 2.0 mL of the extract was combined with 0.5 mL of 5% NaNO_2_ solution. After 6 minutes, 0.5 mL of 10% Al(NO_3_)_3_ solution was added and thoroughly mixed. Following an additional 6 minutes, 4 mL of 4% NaOH solution was added, and the mixture was diluted to volume with 60% ethanol. The solution was homogenized and allowed to stand for 15 minutes before Shimadzu UV-1800 spectrophotometric measurement at 510 nm. Time-course absorbance measurements were initiated upon the addition of 5% NaNO_2_ solution. The flavonoid content in rice flag leaves was determined using 1 mL aliquots of sample solution in 10 mL volumetric flasks.

Total flavonoid content was calculated using the following equation:


Total flavonoid content (mg/g)=(C×N×Vt)/(m×Vs)×0.001

Where: C = flavonoid content derived from the standard curve (μg); N = dilution factor; Vt = total extract volume (mL); Vs = measurement volume (mL); m = sample mass (g).

### 4.5 Transcriptomic analysis of rice leaf samples

Total ribonucleic acid isolation from rice leaf samples was accomplished using the Trizol extraction method (Takara kit). Sequencing libraries were prepared utilizing the UltraTM RNA Library Prep Kit following standard manufacturer guidelines (NEB, USA). Paired-end sequencing was performed on the Illumina HiSeq 2500 sequencing platform. After eliminating adapter contamination and filtering poor-quality sequences, high-quality reads were aligned to the rice reference genome assembly (IRGSP-1.0_genome.fa, retrieved from https://rapdb.dna.affrc.go.jp/index.html on April 11, 2025). The transcriptomic sequencing datasets generated in this study have been archived and made publicly accessible through the China National GeneBank (CNGB; https://db.cngb.org/cnsa) repository and can be retrieved using the project identifier CNP0007364. Functional annotation of genes was conducted through interrogation of multiple reference databases using established bioinformatics pipelines. Raw sequencing data quality control was executed using FastQC software (Brown et al., 2017), while adapter sequences were removed using Cutadapt tool (Martin, 2011). Clean reads were subsequently mapped to the reference genome using the HISAT2 alignment algorithm. Identification of differentially expressed transcripts (DETs) was performed using Cuffdiff analysis with statistical cutoffs of |Log2FoldChange| ≥1 and P-value< 0.05. A comprehensive list of all differentially expressed root genes is available in **Supplementary Table S4**. Functional enrichment analysis of DETs was executed following standard computational protocols (Zheng and Wang, 2008), with KOBAS bioinformatics platform employed to assess statistical significance of enriched pathways among the identified gene sets.

### 4.6 Rice leaf flavonoid profiling

Ultra-performance liquid chromatography coupled with tandem mass spectrometry (UPLC-MS/MS) was utilized to evaluate the flavonoid profiling of rice flag leaves. Preparation of samples involved homogenization of freeze-dried tissue into fine powder, from which 50 mg portions were selected for flavonoid extraction using 500 μL of a solution comprising 50% methanol and 0.1% HCl, with rutin used as an internal standard. The mixture underwent sonication for 30 minutes, followed by centrifugation (12,200×g, 5 minutes) at room temperature. The resulting supernatant was filtered through a 0.22 μm membrane filter prior to analysis. UPLC-MS/MS was executed according to previously established methodologies (Chen et al., 2013). Flavonoids were identified by comparing precursor ions (Q1), product ions (Q3), characteristic fragmentation patterns, and retention times with authentic reference standards analyzed under identical conditions. All measurements were performed in triplicate, with data processing conducted using Analyst 1.6.3 software. A detailed description of all identified flavonoids is available in **Supplementary Table S1**.

### 4.7 Total chlorophyll contents (mg/g)

Leaf samples from rice plants (control, 10%, 30%, and 50% treatments) were evaluated in triplicate to determine total chlorophyll content (Sumanta et al., 2014). For each analysis, 0.2 g of fresh leaf tissue was homogenized in a mortar containing quartz sand, calcium carbonate powder, and 10 mL of 95% ethanol. During the grinding process, an additional 5 mL of 95% ethanol was incorporated until complete decolorization of the tissue was achieved. Following a 3–5-minute settling period, the homogenate was filtered into a 25 mL amber volumetric flask. The residual material on the filter paper was washed with 95% ethanol, and the solution was adjusted to a final volume of 25 mL with ethanol. Spectrophotometric measurements of the extract were conducted at wavelengths of 665 nm and 649 nm, with 95% ethanol serving as the blank reference. Chlorophyll concentrations were subsequently determined using the following formulae:


Chlorophyll a (mg/g fresh weight)=Chlorophyll a×V×N/(W×1000)

Chlorophyll b(mg/g fresh weight)=Chlorophyll b×V×N/(W×1000)

Total chlorophyll (mg/g fresh weight)=Chlorophyll a+Chlorophyll b

Where: V = extract volume (mL) and N = dilution factor.

### 4.8 Statistical analysis

Multivariate statistical analyses were conducted on metabolomic datasets. Data normalization was implemented prior to performing HCA, utilizing Z-score for metabolomic data matrices. Statistical assessments encompassed HCA and PCA following standardized methodological protocols as described by (Chanana et al., 2020). Variable Importance in Projection (VIP) scores were calculated from orthogonal partial least squares-discriminant analysis (OPLS-DA) models using the MetaboAnalystR package in R (Chong and Xia, 2018). Metabolites with VIP scores >1 were considered important contributors to group separation (Thévenot et al., 2015). Differential metabolites were identified based on (1) significance thresholds (P-value<0.05 from Student’s t-tests, with false discovery rate [FDR] correction for multiple comparisons) (Chen et al., 2013), and (2) magnitude thresholds (absolute log2 fold-change [Log2FC] ≥1, corresponding to fold-change [FC] ≥2 or ≤0.5) (Fraga et al., 2010). The details, including preprocessing steps and validation procedures, are provided in **Supplementary File 1**. The statistical significance of variations observed in physiological parameters was assessed using the least significant difference (LSD) test. Standard error calculations and comprehensive statistical analyses were executed using Statistix software version 8.1 (Tallahassee, FL, USA), with all experimental procedures conducted in triplicate to ensure reliability of results. Correlation analyses in the heatmap (**Figure 6**) were performed using Pearson’s correlation coefficient to assess linear relationships between transcription factor expression and flavonoid abundance. Venn diagrams were constructed to visualize overlaps in differentially expressed metabolites between treatment vs control groups using the online tool EVenn (http://www.ehbio.com/test/venn/#/).

## 5 Conclusion

This study elucidates the mechanisms by which *Leucocalocybe mongolica* strain LY9 enhances rice growth and modulates flavonoid metabolism via transcriptional regulation. LY9 treatment significantly improved key agronomic traits, including tillering, shoot and root elongation, and chlorophyll content, while reducing total flavonoid levels, indicative of a growth-defense trade-off. Transcriptomic analyses highlighted the pivotal roles of MYB, bHLH, and WRKY transcription factors, particularly Os04g0605100-WRKY68 and Os05g0553400-R2R3MYB84, in mediating these responses. Metabolomic data revealed selective induction of chalcones and isoflavones, which may compensate for suppressed flavones in stress adaptation. The strong negative correlations between flavonoid content and growth parameters further support LY9’s ability to reallocate resources toward growth under favorable conditions. These findings position LY9 as a promising biofertilizer for sustainable rice cultivation, capable of boosting yield while modulating stress-responsive pathways. Future research should explore field applications and long-term effects of LY9 on soil health and crop resilience, paving the way for eco-friendly agricultural innovations.

## 6 Patents

Currently, there are two pending patent applications relevant to the work in this manuscript. The patent application numbers are 202410517649.4 and 202410517671.9, are submitted to the National Intellectual Property Administration of China. These applications are in line with the innovative aspects of our research and reflect our efforts to protect the intellectual property stemming from this study.

## Data Availability

The raw data of RNA-seq are deposited in China National GeneBank (CNGB; https://db.cngb.org/cnsa) under project accession No. CNP0007364.

## Supplementary material

Supplementary Table 1 Raw Flavonoid data.

Supplementary Table 2 Flavone HCA, F1-F10 names consistent to **Figure 3A**.

Supplementary Table 3 Other flavonoids HCA, F1-F17 names corresponding to **Figure 3B**.

Supplementary Table 4 Raw MYB, bHLH, and WRKY transcriptional factor genes expression patterns.
