# Supplementary material for: Leucocalocybe mongolica inoculation enhances rice growth by reallocating resources from flavonoid defense to development via MYB/bHLH/WRKY networks
Source: Front Plant Sci. 2025 Sep 3;16:1653445. doi: 10.3389/fpls.2025.1653445 (PMC12442037; doi:10.3389/fpls.2025.1653445)
Supplement: Supplementary file 3 [file DataSheet1.docx]

**Standardized Protocol for Cultivation of LY9 Strain on Potato Dextrose Agar (PDA) Medium**

**1. Preparation of PDA**

**1.1 Component Preparation and Initial Processing**

The preparation begins with precise quantification of media constituents following standardized formulations. Potatoes undergo mechanical processing (peeling and uniform dicing) before being transferred to a sterile receptacle. Subsequently, 1000 mL of distilled water is introduced to the processed potato material in an appropriate vessel and heated to its boiling point. The mixture is maintained at this temperature for a 20–30-minute interval to facilitate optimal extraction of starch components. Following the thermal extraction process, the heated mixture undergoes filtration through sterilized gauze, with careful retention of the filtration. The filtered extract is then volumetrically adjusted with additional distilled water to achieve a final volume of 1000 mL.

**1.2 Medium Formulation and Sterilization**

The potato extract is supplemented with 20 g of glucose and 15-20 g of agar. The suspension is positioned on an asbestos net and subjected to controlled heating while undergoing continuous agitation with a glass rod until complete solubilization of the agar component is observed. The homogeneous medium is then aliquoted into conical flasks and subjected to standard autoclave sterilization protocols (121°C, 15 minutes).

**2. LY9 Inoculation Methodology**

Under controlled aseptic conditions within a laminar flow environment, a sterilized inoculation instrument is utilized to excise a small mycelial segment from a viable LY9 culture. This fungal propagule is aseptically transferred to the prepared PDA substrate, ensuring direct contact between the mycelial surface and the nutritive medium at a predetermined position. Each culture vessel is appropriately identified and sealed to maintain aseptic integrity.

**3. Incubation Parameters**

The inoculated cultures are maintained at a constant temperature of 25°C for a duration of 20 days. This incubation period facilitates comprehensive colonization of the substrate surface by the fungal mycelia, at which point the cultures attain optimal development for subsequent experimental applications.

**Methods for Calculating VIP Scores, P-values, Fold Changes, and Log2FC in Metabolomics Analysis**

1. **Variable Importance in Projection (VIP) Scores**

VIP scores were calculated using the Orthogonal Partial Least Squares-Discriminant Analysis (OPLS-DA) model. The OPLS-DA analysis was performed using the R package MetaboAnalystR (Chong & Xia, 2018). The model decomposes the predictor matrix (X) into components correlated (predictive) and uncorrelated (orthogonal) to the response matrix (Y). VIP values quantify the contribution of each metabolite to the separation of groups in the OPLS-DA model. Metabolites with VIP > 1 were considered statistically significant for group discrimination (Thévenot et al., 2015).

**Reference**:

- Thévenot, E. A., Roux, A., Xu, Y., et al. (2015). Analysis of the Human Adult Urinary Metabolome Variations with Age, Body Mass Index, and Gender by Implementing a Comprehensive Workflow for Univariate and OPLS Statistical Analyses. *Journal of Proteome Research*, 14(8), 3322–3335.

2. **P-values**

P-values were derived from univariate statistical tests (e.g., Student’s t-test or Welch’s t-test for two-group comparisons) to assess the significance of differences in metabolite levels between groups. The false discovery rate (FDR) was calculated using the Benjamini-Hochberg method to correct for multiple testing (Chen et al., 2013).

**Reference**:

- Chen, W., Gong, L., Guo, Z., et al. (2013). A Novel Integrated Method for Large-Scale Detection, Identification, and Quantification of Widely Targeted Metabolites: Application in the Study of Rice Metabolomics. *Molecular Plant*, 6(6), 1769–1780.

3. **Fold Change (FC) and Log2 Fold Change (Log2FC)**

FC was calculated as the ratio of the mean metabolite intensity in the experimental group to that in the control group:

FC=Mean intensity (Experimental group)/Mean intensity (Control group)

FC=Mean intensity (Control group)Mean intensity (Experimental group)​

Log2FC was computed as the base-2 logarithm of FC:

Log2FC=log_2_(FC)

Metabolites with |Log2FC| ≥ 1 (i.e., FC ≥ 2 or ≤ 0.5) were considered differentially abundant (Fraga et al., 2010).

**Reference**:

- Fraga, C. G., et al. (2010). Signature-discovery Approach for Sample Matching of a Nerve-Agent Precursor Using Liquid Chromatography-Mass Spectrometry, XCMS, and Chemometrics. *Analytical Chemistry*, 82(10), 4165–4173.

4. **Statistical Workflow**

- **Data Preprocessing**: Raw data were log2-transformed and mean-centered before OPLS-DA. Missing values were imputed using the k-nearest neighbors (KNN) algorithm.
- **Model Validation**: Permutation tests (200 iterations) were performed to evaluate OPLS-DA model robustness (Q2 > 0.5 considered valid).
- **Integration**: Differential metabolites were selected based on VIP > 1, |Log2FC| ≥ 1, and FDR-adjusted P-value < 0.05.

**Reference**:

- Chong, J., & Xia, J. (2018). MetaboAnalystR: An R Package for Flexible and Reproducible Analysis of Metabolomics Data. *Bioinformatics*, 34(24), 4313–4314.


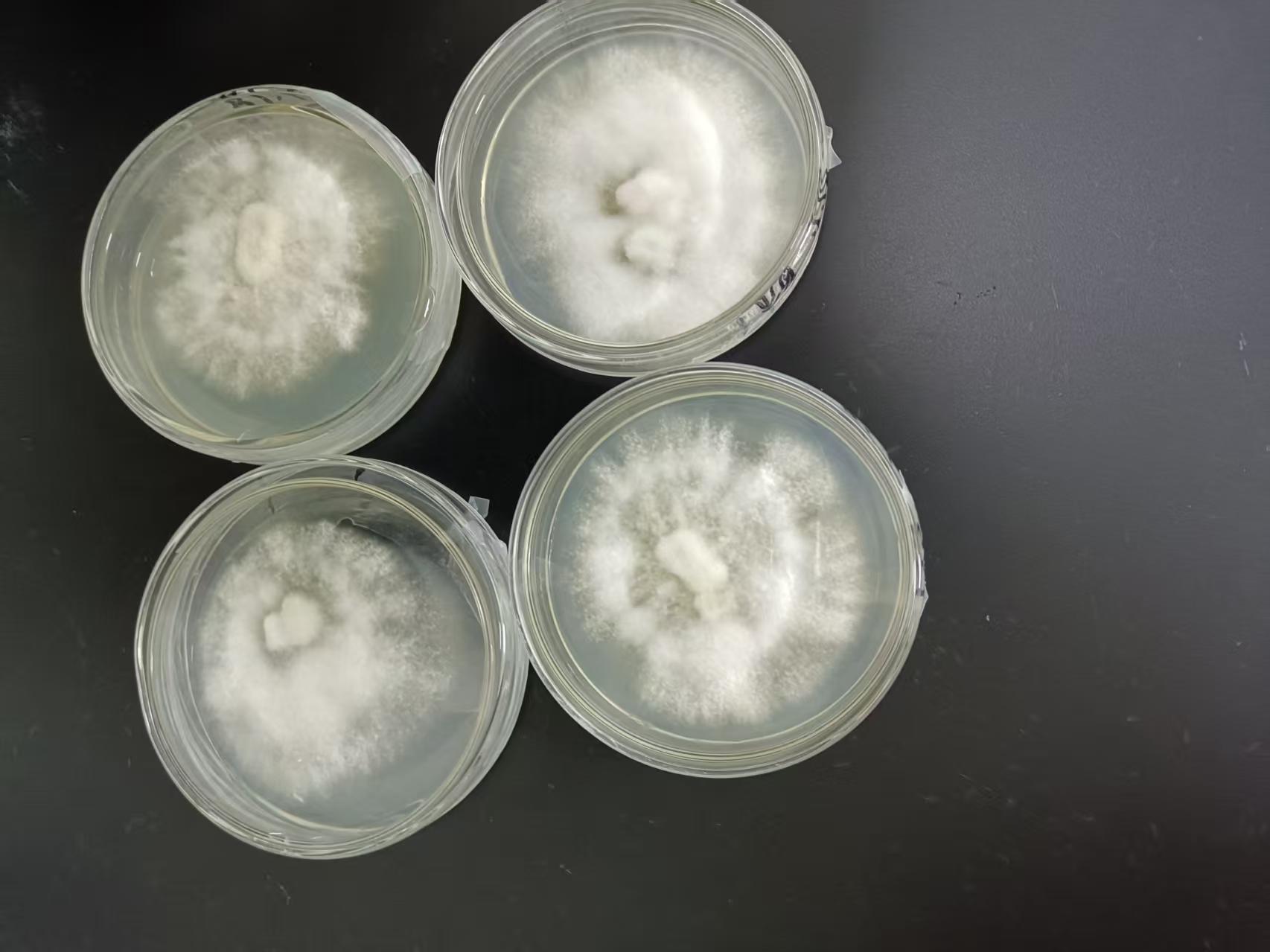


**Supplementary Figure S1** depicts the Potato Dextrose Agar (PDA) medium exhibiting successful colonization by the LY9 strain.


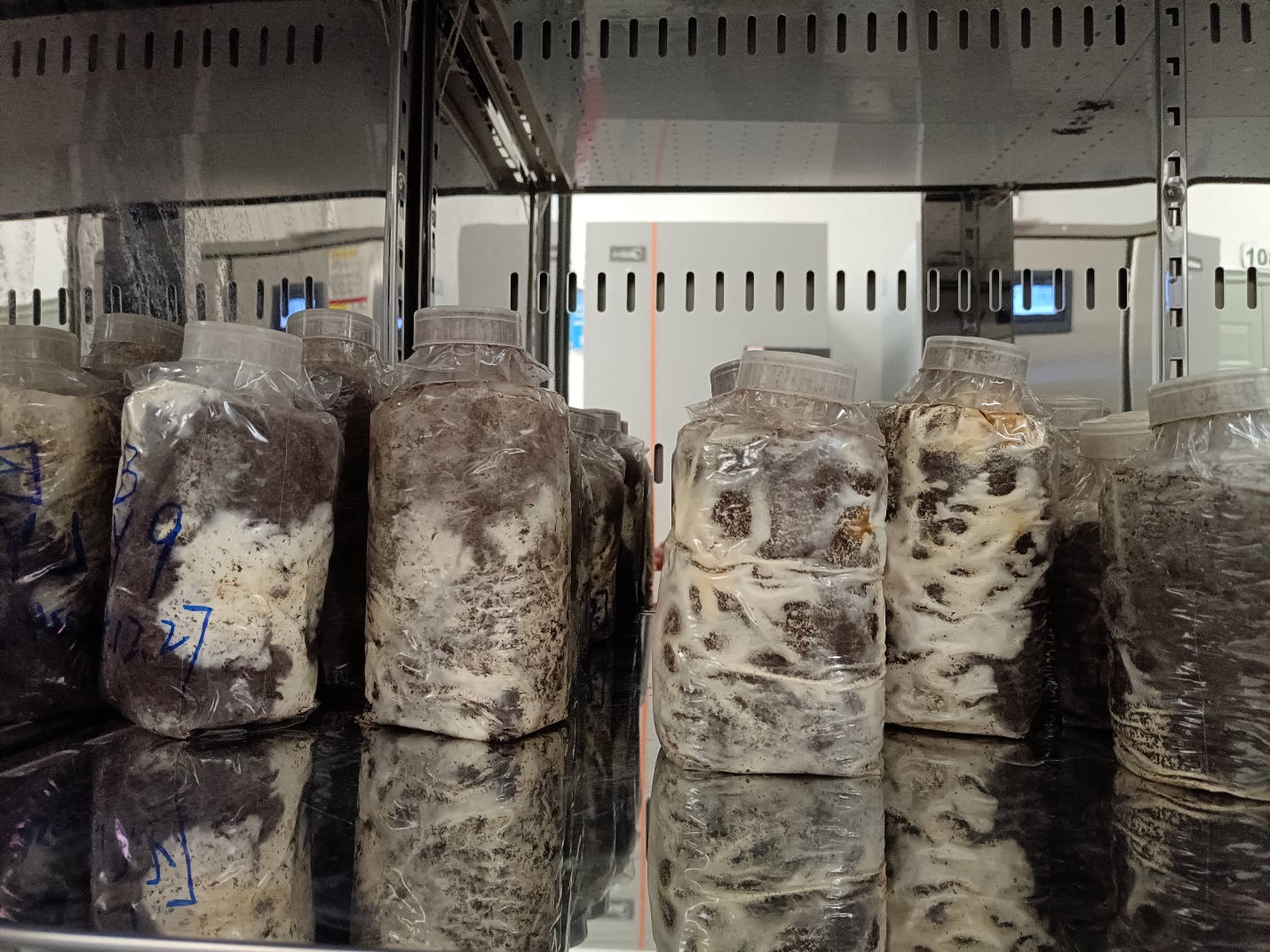


**Supplementary Figure S2** presents the prepared LY9-transformed soil culture alongside the characteristic surface features of the LY9-transformed soil matrix.
